# A Quenched Annexin V‐Fluorophore for the Real‐Time Fluorescence Imaging of Apoptotic Processes In Vitro and In Vivo

**DOI:** 10.1002/advs.202002988

**Published:** 2020-10-28

**Authors:** Hyunjin Kim, Hee Yeon Kim, Eun Young Lee, Boem Kyu Choi, Hyonchol Jang, Yongdoo Choi

**Affiliations:** ^1^ Research Institute National Cancer Center 323 Ilsan‐ro Goyang Gyeonggi 10408 Republic of Korea; ^2^ Department of Cancer Biomedical Science National Cancer Center Graduate School of Cancer Science and Policy 323 Ilsan‐ro Goyang Gyeonggi 10408 Republic of Korea

**Keywords:** 3D cells, annexin V, in vivo, off‐on type, real‐time apoptosis imaging

## Abstract

Annexin‐based probes have long been used to study apoptotic cell death, which is of key importance to many areas of biological research, drug discovery, and clinical applications. Although apoptosis is a dynamic biological event with cell‐to‐cell variations, current annexin‐based probes are impractical for monitoring apoptosis in real‐time. Herein, a quenched annexin V‐near‐infrared fluorophore conjugate (Q‐annexin V) is reported as the first OFF‐ON annexin protein‐based molecular sensor for real‐time near‐infrared fluorescence imaging of apoptosis. Q‐annexin V is non‐fluorescent in the extracellular region, due to photoinduced electron transfer interactions between the conjugated dye and amino acid quenchers (tryptophan and tyrosine). The probe becomes highly fluorescent when bound to phosphatidylserines on the outer layer of cell membranes during apoptosis, thereby enabling apoptosis to be monitored in real‐time in 2D and 3D cell structures. In particular, Q‐annexin V shows superior utility for in vivo apoptosis fluorescence imaging in animal models of cisplatin‐induced acute kidney injury and cancer immune therapy, compared to the conventional polarity‐sensitive pSIVA‐IANBD or annexin V‐Alexa647 conjugates.

## Graphical abstract

A quenched annexin‐V near‐infrared fluorophore (Q‐annexin V) conjugate is developed as the first OFF‐ON annexin protein‐based molecular sensor for real‐time near‐infrared fluorescence imaging of apoptosis. Q‐annexin V becomes highly fluorescent when bound to phosphatidylserine on the outer layer of cell membranes during apoptosis, thereby enabling apoptosis to be monitored in real‐time in vitro and in vivo.

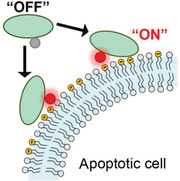

## 1 Introduction

Apoptosis is a form of programmed cell death that plays a critical role in organism development and various diseases, including cancer.^[^ ^1^, ^2^ ^]^ Anti‐cancer therapies eliminate tumor cells via apoptosis, and the dysregulation of various genes regulating apoptosis has been strongly associated with resistance to anti‐cancer therapy.^[^ ^2^ ^]^ It is important to monitor apoptosis to appropriately evaluate therapeutic efficacy. Various methods have been developed to detect apoptosis, most of which are unsuitable for kinetic apoptosis detection.

Due to the different vulnerabilities of individual cells, there are intercellular differences in the timing and severity of their responses to chemical drugs and apoptotic stimuli. In cancer, cells that do not undergo apoptosis during drug treatment are one of the main causes of poor prognosis. To identify and study ways to regulate these drug‐resistant cells, it is critical to monitor the response of individual cells in real time. Recently, 3D cell cultures such as tumor spheroid and organoid cultures have been regarded as more physiological preclinical cancer models, and their use in cancer research has rapidly increased.^[^ ^3^, ^4^ ^]^ Therefore, it is necessary to develop a method for observing apoptosis in individual cells under 3D culture conditions. More importantly, the efficacy of cancer immunotherapy, which has recently received attention, can be appropriately evaluated only through animal experiments,^[^ ^5^, ^6^ ^]^ and for effective evaluation, a tool capable of monitoring apoptosis in an animal model in real‐time is needed.

Annexin proteins have been widely used to analyze cellular apoptotic events by specifically detecting phosphatidyl serine (PS) exposure on the outer leaflet of the cellular membrane during the early stage of apoptosis.^[^ ^7^ ^]^ The standard protocols of apoptosis assays using currently available annexin‐based probes (e.g., annexin protein‐fluorophore conjugates) consist of a multi‐step process: 1) cells are treated with the probes for a certain period of time; 2) cells are washed to remove background signals from unbound probes; and 3) fluorescence imaging is performed using microscopes. In 2D culture, cells that undergo apoptosis are generally weakly attached and can be easily detached during the washing step. In 3D culture, it is difficult to remove unbound annexin‐based probes inside 3D cells such as spheroids and organoids. Thus, currently available annexin‐based probes are impractical for monitoring apoptosis in real time or in 3D culture because of the inevitable washing step and have limitations for detecting dynamic and individual apoptotic events in 2D and 3D cultures.

To overcome these problems, Langen's group engineered annexin BXII, which has cysteine mutations at residues 101 and 260 in membrane‐binding loops, and then conjugated a polarity‐sensitive dye (with excitation and emission peaks at 488 and 530 nm, respectively) to the engineered annexin protein.^[^ ^8^ ^]^ The polarity‐sensitive annexin BXII‐fluorophore conjugate is nonfluorescent (OFF) in polar aqueous environments (i.e., extracellular region) but becomes highly fluorescent (ON) after binding to the membrane of an apoptotic cell, thereby enabling apoptotic cell death imaging in real‐time. However because this method uses polarity‐sensitive dyes with short excitation and emission wavelengths, the light penetration depth is limited, and it is therefore difficult to monitor apoptotic events that occur in deeply seated biological tissues in vivo. No new OFF/ON near‐infrared (NIR) fluorescence probes have yet been reported for monitoring apoptotic events in real‐time in vivo; therefore, the study of this process remains an ongoing challenge.

**Figure 1:**
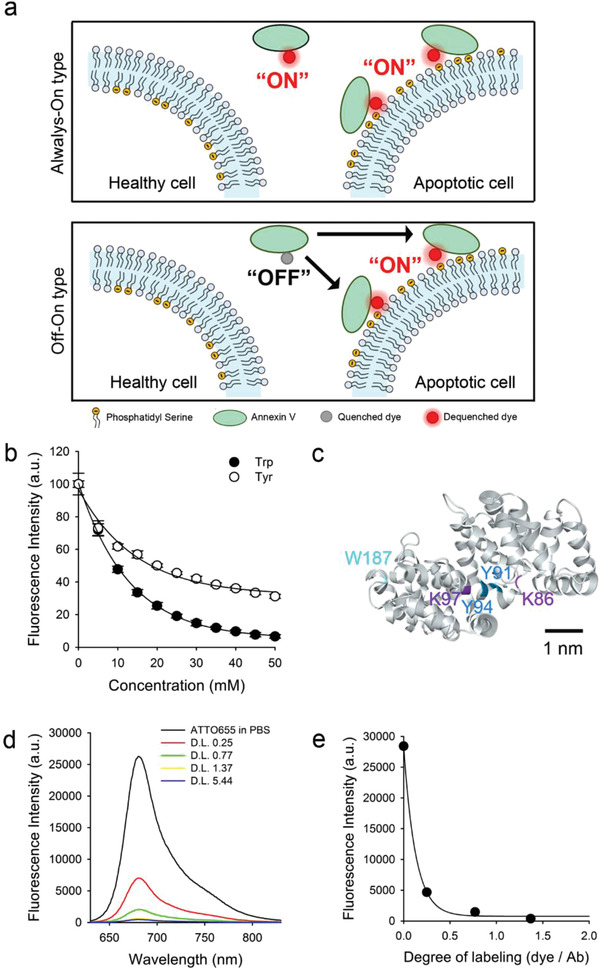
a) Schematic diagram of selective and real‐time fluorescence imaging of apoptotic cells using OFF/ON type annexin V‐fluorophore conjugate (Q‐annexin V). b) Fluorescence quenching of ATTO655 (1 × 10^−6^ m) in the presence of various tryptophan (Trp) and N‐acetyl‐L‐tyrosine (Tyr) concentrations (*n* = 3): *λ*_ex_ 663 nm, *λ*_em_ 684 nm. c) 3D structure of annexin V (1AVH) from Web3DMol. Lysine residues (Lys86 and Lys97) for dye conjugation and amino acid quenchers (Trp187, Tyr91, and Tyr94) are located within 1 nm for effective PET‐based quenching. d) Comparison of fluorescence spectra (*λ*_ex_ 600 nm *λ*_em_ 610–850 nm) for Q‐annexin V with different degree of labeling (DL) and free ATTO655 dye at an equimolar dye concentration (i.e., 1 × 10^−6^ m dye equivalent). e) Correlation between DL and fluorescence intensity (*λ*_ex_ 600 nm, *λ*_em_ 684 nm).

Herein, we developed a quenched annexin V‐NIR fluorophore conjugate (Q‐annexin V) as a new OFF/ON type fluorescence sensor for real‐time monitoring of apoptosis (**Figure** **1**a). The fluorescence of Q‐annexin V is quenched (OFF) by photoinduced electron transfer (PET) interactions between the conjugated NIR fluorophore and amino acid quenchers (tryptophan and/or tyrosine) in close proximity (0.5–1.5 nm).^[^ ^9^ ^]^ In this study, the zwitterionic NIR fluorophore ATTO655, which has good fluorescence quantum yield and excellent photostability, was used to synthesize Q‐annexin V as the dye showed high fluorescence quenching efficacy by amino acid quenchers (tryptophan and tyrosine) and is considered an excellent PET‐based fluorescence sensor for assessing small conformational changes in proteins.^[^ ^10^, ^11^, ^12^, ^13^ ^]^ The calcium ion (Ca^2+^)‐mediated binding of annexin V to anionic phospholipid membranes induces significant conformational changes in annexin V, and tryptophan (and/or tyrosine)‐induced PET quenching is highly sensitive for assessing small changes in protein structure.^[^ ^14^, ^15^, ^16^, ^17^, ^18^ ^]^ Thus, we hypothesized that the instantaneous change in protein structure when Q‐annexin V binds exposed PS on the outer surface of early apoptotic cells would change the distance between the conjugated fluorophore and tryptophan (and/or tyrosine), resulting in the fluorescence dequenching (ON) of Q‐annexin V. This OFF/ON behavior of Q‐annexin V upon its binding to cells with exposed PS enables early apoptotic events to be monitored in real‐time in vitro and in vivo.

## 2 Results

### 2.1 Q‐Annexin V Synthesis and Characterization

First, we tested the fluorescence quenching effect of the ATTO655 dye by amino acid quenchers. When ATTO655 was mixed with tryptophan or tyrosine, its fluorescence was efficiently quenched in an aqueous solution with increasing concentrations of added tryptophan or tyrosine (Figure 1b). Since there was no overlap between the fluorescence spectrum of the dye and the absorption spectrum of these amino acids, the dye was quenched via PET interactions between ATTO655 and the amino acids in close proximity, as reported previously.^[^ ^9^, ^10^, ^19^, ^20^ ^]^

Conventional NHS chemistry was used to synthesize Q‐annexin V (i.e., the annexin V‐ATTO655 conjugate). Briefly, ATTO655‐NHS esters were reacted with the amine group of the annexin V lysine residues at various reaction ratios (Figure 1c). The conjugate was purified by passing the solution through a Sephadex G25 gel filtration column to remove unreacted dyes and byproducts. The number of conjugate dyes per annexin V molecule (i.e., degree of labeling, DL) was analyzed by measuring the absorbance of the conjugates. When the DL versus the fluorescence spectrum of the prepared samples was analyzed (Figure 1d,e), a dramatic quenching effect was observed at a DL of 0.25, compared with free dye at an equimolar dye concentration, again suggesting that fluorescence quenching occurred via PET interactions between the dye and amino acid quenchers, not via Förster resonance energy transfer (FRET) between the conjugate dyes. The fluorescence intensity of the conjugated dye was further reduced at higher DL values (i.e., 0.77 and 1.37).

When Q‐annexin V was treated with a denaturation solution containing a surfactant and reducing agent (1 w/v % sodium dodecyl sulfate + 1 × 10^−3^ m 2‐mecaptoethanol) to disintegrate its 3D structure, an 18.8‐fold increase in its fluorescence was observed (Figure S1a, Supporting Information). In contrast, when annexin V‐Alexa647, a commercially available agent used to analyze apoptotic events by flow cytometry, was treated with the denaturation solution, only a 1.4‐fold increase was observed (Figure S1b, Supporting Information). These data indicate that changes in the distance between conjugated ATTO655 and the amino acid quenchers induces the fluorescence of the dye to be turned on.

### 2.2 Stability of Quenched Q‐Annexin V

We determined whether the presence of serum proteins affected Q‐annexin V quenching. The conjugate was incubated in either phosphate‐buffered saline solution (PBS, pH 7.4) or PBS containing 10% human serum albumin (HSA), and the change in fluorescence intensity over time was monitored. No change in Q‐annexin V fluorescence intensity was observed during the 72 h of incubation (Figure S2, Supporting Information) in either PBS or HSA‐containing PBS, indicating that there was no interaction between the conjugated dye and HSA. Consequently, the PET interactions between the conjugated dye and the amino acid quenchers were preserved.

### 2.3 Reaction of Q‐Annexin V with Phosphatidylserine (PS)‐Presenting Liposomes

**Figure 2:**
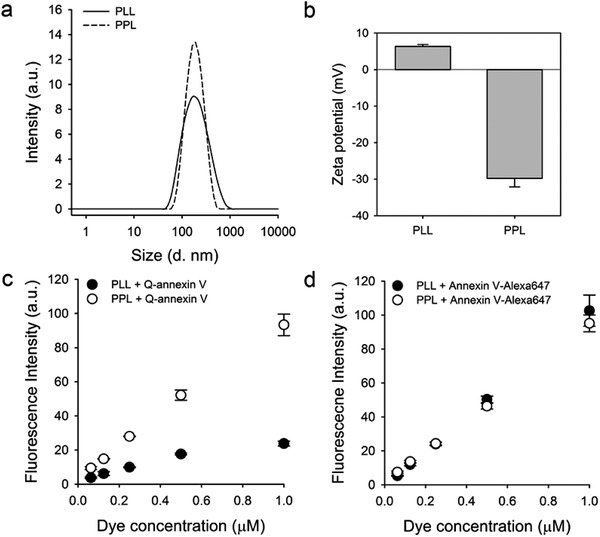
Fluorescence turn‐on upon binding with phosphatidylserine‐presenting liposomes (PPL). a) Hydrodynamic size and b) zeta potential of PS‐lacking liposomes (PLLs) and PPLs in an aqueous solution (*n* = 3). c) Fluorescence turn‐on of Q‐annexin V when mixed with PLLs and PPLs with increasing concentrations of Q‐annexin V (*n* = 3). Q‐annexin V fluorescence was increased by treating with PPLs, indicating the turn‐on of its quenched fluorescence. d) Fluorescence changes of annexin V‐Alexa647 in the presence of PLLs and PPLs for comparison (*n* = 3).

It was expected that the fluorescence of Q‐annexin V could be turned on upon binding with PS on the apoptotic cell membrane; therefore, we measured the fluorescence intensity of Q‐annexin V after treatment with PS‐presenting liposomes (PPL), an in vitro model system that mimics apoptotic cell membranes.^[^ ^21^ ^]^ PS‐lacking liposomes (PLLs) were used as controls. The hydrodynamic sizes of the prepared PLLs and PPLs were 172 ± 76.65 and 167.3 ± 40.26 nm (**Figure** **2**a), and their zeta potentials were 6.33 ± 0.49 and −29.8 ± −2.34 mV, respectively (Figure 2b). Q‐annexin V and annexin V‐Alexa647 were mixed with the liposomes in a reaction buffer containing Ca^2+^. The fluorescence intensity of Q‐annexin V increased by approximately fourfold when incubated with PPLs compared to PLLs (Figure 2c); however, the fluorescence of annexin V‐Alexa647 did not change when incubated with PPLs or PLLs as annexin V‐Alexa647 was not quenched (Figure 2d). Therefore, PPL binding did not affect the fluorescence of the control probe.

### 2.4 Real‐Time Imaging of Apoptotic Cells with Q‐Annexin V

**Figure 3:**
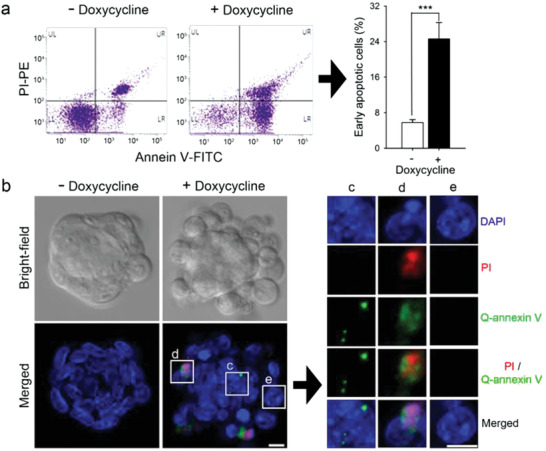
Q‐annexin V can detect apoptotic cells without washing. a) Conventional detection of apoptotic cells by flow cytometry. ZHBTc4 mouse embryonic stem cells (mESCs) were depleted of Oct4 by treatment with doxycycline (Dox) for 3 days. Apoptotic cells were quantified by flow cytometry (*n* = 3) after staining with annexin V‐FITC and propidium iodide (PI), ***P < 0.001. b) Q‐annexin V, PI, and 4’,6‐diamidino‐2‐phenylindole (DAPI) were added to the ZHBTc4 cells treated with or without Dox for 3 days. Cells were imaged without washing by confocal microscopy (LSM 510; Carl Zeiss). c–e) A representative image of mESC colonies and representative enlarged images of cells at various stages of apoptosis are shown in (c–e). c) Early apoptotic cells only positive for Q‐annexin V. d) late apoptotic cells positive for both Q‐annexin V and PI. e) non‐apoptotic cells negative for both Q‐annexin V and PI.

Before assessing the potential of Q‐annexin V for real‐time monitoring of apoptosis, its ability to detect apoptotic cells in the absence of a washing step was tested in the context of differentiation‐associated apoptosis. In mouse embryonic stem cells (mESCs), the removal of Oct4, a major transcription factor that controls pluripotency, results in differentiation and eventually significant levels of apoptosis.^[^ ^22^ ^]^ By conventional flow cytometry‐based apoptosis analysis, depleting Oct4 with doxycycline (Dox) in ZHBTc4 mESCs^[^ ^23^ ^]^ increased the number of early apoptotic cells (annexin V‐positive and propidium iodide (PI)‐negative cells) by ≈20% (**Figure** **3**a). For the image‐based apoptosis analysis, Q‐annexin V and PI were added to the culture media of Dox‐treated and non‐treated ZHBTc4 cells and the cells were imaged by confocal microscopy without washing. Dox‐treated cells showed an apparent increase in Q‐annexin V and PI signals compared to the untreated cells (Figure 3b). In addition, cells in various stages of apoptosis were detected; early apoptotic cells were only positive for Q‐annexin V, late apoptotic cells were positive for both Q‐annexin V and PI, and non‐apoptotic cells were negative for both Q‐annexin V and PI (Figure 3b).

**Figure 4:**
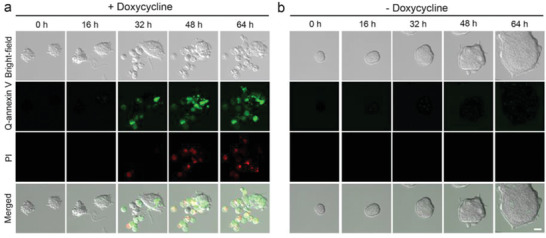
Detection of apoptotic cells in real time. a,b) ZHBTc4 cells treated with or without Dox for 2 days were treated with both Q‐annexin V and PI and imaged by confocal microscopy (LSM 780; Carl Zeiss) at 30 min intervals for 3 days. Representative images are shown. Full movies are presented in Movie S1 and S2 (Supporting Information). Scale bar = 20 µm.

Next, we monitored cell apoptosis in real‐time. ZHBTc4 cells were treated with or without Dox for 2 days. After Q‐annexin V and PI were added, cells were imaged using a confocal microscope (*λ*_ex_ 633 nm, *λ*_em_ 647–754 nm for Q‐annexin V) at 30 min intervals for three days in real‐time without washing. Oct4 depletion led to the morphological differentiation of ZHBTc4 cells, which eventually underwent apoptosis, as indicated by the increase in Q‐annexin V‐positive cells. Q‐annexin V‐positive cells later became PI positive (**Figure** **4**a and Movie S1, Supporting Information). The control cells without Dox‐treatment continued to be negative for both Q‐annexin V and PI (Figure 4b and Movie S2, Supporting Information). These results suggest that Q‐annexin V could be used to follow apoptotic cells in real‐time for at least 3 days.

### 2.5 Apoptosis Imaging in 3D Cell Culture Models

**Figure 5:**
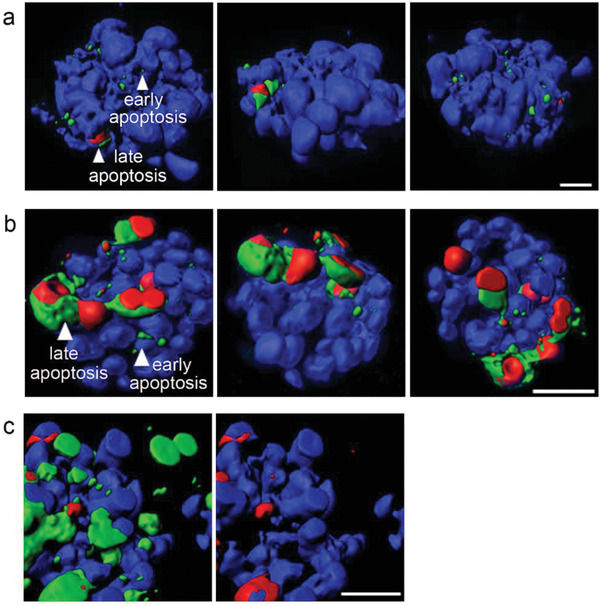
Real‐time apoptosis imaging in 3D cell culture systems. a) ZHBTc4 cells were differentiated by embryoid body (EB) formation. 3D EBs were stained with Q‐annexin V, PI, and DAPI. b) 3D tumor spheroids of H358 lung cancer cells were treated with cisplatin (20 × 10^−6^ m) for 24 h and stained with Q‐annexin V, PI, and Hoechst33342. Without washing the cells, Z‐stack images were obtained, and 3D images were produced by confocal microscopy (LSM 510; Carl Zeiss). Surface rendered 3D images were obtained using imaris (Bitplane). Blue: DAPI or Hoechst33342, Red: PI, Green: Q‐annexin V. c) Difficulties in detecting apoptotic cells in 3D cell culture systems using a conventional always‐on Probe. 3D tumor spheroids of H358 lung cancer cells treated with cisplatin were incubated with annexin V‐Alexa647, PI, and Hoechst33342. Without washing the cells, Z‐stack images were obtained by confocal microscopy and surface rendered 3D images were obtained using imaris (Blue: Hoechst33342, Red: PI, Green: annexin V‐Alexa647). Left: merged fluorescence image of Hoechst33342, PI, and annexin V‐Alexa647, Right: merged fluorescence image of Hoechst33342 and PI is shown for comparison. Scale bar = 20 µm.

The utility of Q‐annexin V was validated in 3D cell culture systems. Q‐annexin V, PI, and DAPI were added to a 3D embryoid body (EB) derived from ZHBTc4 cell differentiation in a non‐adherent culture dish.^[^ ^24^ ^]^ Confocal images were obtained without washing the cells. Since some EB cells undergo spontaneous apoptosis,^[^ ^25^ ^]^ Q‐annexin V‐positive early apoptotic cells and Q‐annexin V and PI double‐positive late apoptotic cells were detected (**Figure** **5**a and Movie S3, Supporting Information).

Q‐annexin V was also used to detect drug‐induced apoptosis in 3D tumor spheroids. H358 lung cancer tumor spheroids were treated with cisplatin, a primary drug for treating lung cancer, for 24 h and then incubated with Q‐annexin V, PI, and Hoechst33342. A few minutes later, confocal microscopy images were obtained without washing, clearly showing cells undergoing early and late apoptosis (Figure 5b and Movie S4, Supporting Information). These results confirm that Q‐annexin V can be used to follow apoptotic cells in 3D cell culture systems.

For comparison, we performed apoptosis imaging in a 3D tumor spheroid model using a conventional always‐on probe, annexin V‐Alexa647 (Figure 5c and Movie S5, Supporting Information). The cisplatin‐treated tumor spheroids were incubated with annexin V‐Alexa647, PI, and Hoechst33342, and then, without washing the cells, confocal microscopy images were obtained (*λ*_ex_ 633 nm, *λ*_em_ 656–755 nm for annexin V‐Alexa647). As expected, nonspecific strong fluorescence signals were observed in the extracellular regions between H358 cells as well as in the cell‐free space. This result indicates that the always‐on type of annexin V probes is not suitable for real‐time imaging of cell apoptosis in 3D cell culture systems.

### 2.6 In Vivo Apoptosis Imaging in a Cisplatin‐Induced Acute Kidney Injury (AKI) Mouse Model

Next, we evaluated the potential utility of Q‐annexin V for in vivo apoptosis imaging in a drug‐induced AKI mouse model. Cisplatin is a widely used and highly effective drug for the treatment of many cancers, but its clinical use is limited by its nephrotoxicity, including its ability to cause AKI.^[^ ^26^, ^27^ ^]^ In particular, ≈20–40% of patients who receive cisplatin treatment developed dose‐related severe AKI. AKI manifests as a rapid decline in renal function and is a significant health issue. Therefore, the development of NIR fluorescence imaging probes for real‐time diagnosis of apoptosis is of great importance for in vivo screening of new agents that ameliorate drug‐induced AKI.

**Figure 6:**
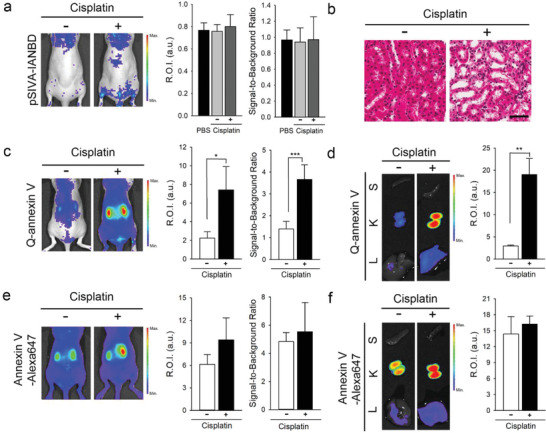
In vivo apoptosis imaging of cisplatin‐induced acute kidney injury (AKI) in a BALB/c‐nu mouse model. The BALB/c‐nu mice treated with or without cisplatin received intravenous injections of annexin‐fluorophore conjugates at a dose of 5 nmol annexin equivalent. After 3 h of the injection of the conjugate, live and ex vivo fluorescence images of a) pSIVA‐IANBD‐, c,d) Q‐annexin V‐, and e,f) annexin V‐Alexa647‐injected mice were obtained. b) Representative hematoxylin and eosin staining of kidney sections of the mice without and with cisplatin treatment. Scale bar = 100 µm. Quantitative analyses of fluorescence intensities of the kidney site (R.O.I) and signal‐to‐background ratio of the kidney were performed using the in vivo fluorescence images (*n* = 3). d,f) Ex vivo images of the spleen (S), kidney (K), and liver (L) (left column), and quantitative analysis of the fluorescence intensity of the kidneys (right column, *n* = 6). *P < 0.05, **P < 0.01, and ***P < 0.001.

BALB/c‐nu mice received an intravenous injection of cisplatin (5 mg kg^−1^) once a day for three days to induce AKI (Figure S3a, Supporting Information).^[^ ^28^ ^]^ Twenty‐four hours after the third cisplatin injection, annexin protein‐fluorophore conjugates (5 nmol annexin protein equivalent in 0.1 mL of PBS) were injected into the mice via the tail vein. Fluorescence imaging was then performed 3 h after the injection of the conjugate. For comparison, the annexin protein‐fluorophore conjugates were also injected into a separate set of control mice that did not receive cisplatin injections. Specifically, PBS‐injected mice that did not receive cisplatin treatment were used as the untreated control group for auto‐fluorescence imaging. When pSIVA‐IANBD, a commercially available polarity‐sensitive annexin BXII‐fluorophore conjugate,^[^ ^8^ ^]^ was injected into the mice, no apparent increase in fluorescence intensity was detected at the kidney sites, regardless of cisplatin treatment or PBS injections (**Figure** **6**a and Figure S4, Supporting Information). Since cisplatin‐induced AKI was confirmed by hematoxylin and eosin (H&E) staining analysis of kidney sections (Figure 6b), the lack of pSIVA‐IANBD fluorescence signals in the kidneys was due to the inherent limitation, that is, the low tissue‐penetration depth of pSIVA‐IANBD fluorescence. As mentioned above, the excitation and emission wavelengths of pSIVA‐IANBD are short (i.e., *λ*_ex_ 460/20 nm and *λ*_em_ 570/40 nm); therefore, this agent was not able to detect cisplatin‐induced AKI, which occurs in a deeply seated location inside the body.^[^ ^29^ ^]^

In contrast, NIR fluorescence images (*λ*_ex_ 640/20 nm and *λ*_em_ 710/40 nm) of Q‐annexin V‐injected mice showed distinct fluorescence emission at kidney sites in the cisplatin‐induced AKI model, compared with mice that did not receive cisplatin treatment (Figure 6c). The mean fluorescence intensity of the kidney sites (R.O.I) in cisplatin‐treated mice was 3.3‐fold higher than that in mice without cisplatin treatment (*P* < 0.05). The signal‐to‐background ratio also increased, from 1.39 ± 0.38 to 3.66 ± 0.73, upon treatment with cisplatin (*P* < 0.001). This finding indicates that the otherwise quenched fluorescence of Q‐annexin V was turned on in the injured kidneys. When we analyzed the ex vivo organ images (Figure 6d and Figure S3b, Supporting Information), the mean fluorescence intensity of the injured kidneys was sixfold higher than that of the healthy kidneys (*P* < 0.01). Ex vivo images of the liver, kidneys, and spleen support the quenching of the fluorescence of Q‐annexin V in healthy kidneys and other organs and the activation of its fluorescence in the injured kidneys.

As expected, NIR fluorescence signals could be detected at the kidney sites in annexin V‐Alexa647‐injected mice (Figure 6e and Figure S3c, Supporting Information). However, no significant differences in the fluorescence intensities of kidneys and signal‐to‐background ratios were observed between mice treated with and without cisplatin, even though the mean values were slightly higher in the cisplatin‐treated mice than in mice without cisplatin treatment (Figure 6e,f). As observed in Figure 5c, the annexin V‐Alexa647 conjugate emitted strong fluorescence in the extracellular space and on the surface of apoptotic cells. Likewise, the generation of strong fluorescence signals from the annexin V‐Alexa647 conjugate in the extracellular space of the kidneys overlaps with the fluorescence signals from the surface of apoptotic kidney cells, thereby limiting the detection of cisplatin‐induced AKI.

### 2.7 In Vivo Apoptosis Imaging in a Cancer Immune Therapy Model

We also corroborated the utility of Q‐annexin V for the early detection of apoptotic events during immunotherapy using immune checkpoint inhibitors (ICIs). To this end, MC38 cells (C57BL6 murine colon adenocarcinoma) were subcutaneously implanted in the right shoulder of C57BL/6 mice. When tumor sizes reached ≈100 mm^3^ on day 10, the mice intraperitoneally received anti‐PD‐1 (0.1 mg/0.1 mL PBS) plus anti‐4‐1BB (0.1 mg/0.1 mL PBS) antibodies 10 and 12 days after tumor implantation (Figure S5, Supporting Information).^[^ ^30^ ^]^ On day 14, Q‐annexin V or annexin V‐Alexa647 was intravenously injected into the mice (n = 4 per group). NIR fluorescence images were obtained 3 h after the injection of the conjugates, using an in vivo imaging system (*λ*_ex_ 640/20 nm, *λ*_em_ 710/40 nm). To analyze fluorescence signals from the tumors and organs in the absence of antibody treatment, a separate set of tumor‐bearing mice received intravenous injections of PBS (0.1 mL; *n* = 4), Q‐annexin V (*n* = 4), and annexin V‐Alexa647 (*n* = 5) on day 10, and in vivo NIR fluorescence images of the mice were obtained 3 h later, as described above. Ex vivo NIR fluorescence images of the tumor, spleen, and kidney in this group were also obtained after in vivo imaging.

**Figure 7:**
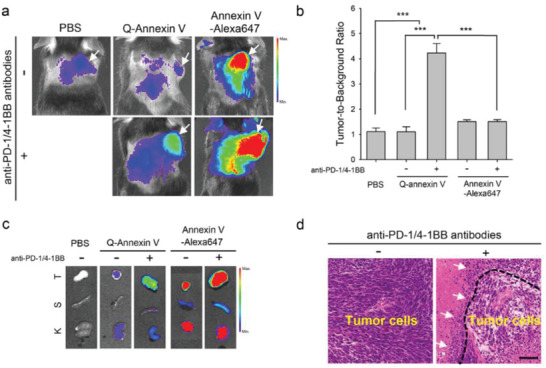
In vivo apoptosis imaging of immune therapy in an MC38 tumor model. a) Representative NIR fluorescence images obtained at 3 h post‐injection of PBS, Q‐annexin V, and annexin V‐Alexa647 in mice with or without anti‐PD‐1/4‐1BB antibody treatment. The arrows indicate the tumor sites. b) Quantitative analysis and comparison of tumor‐to‐background ratios of the groups (*n* = 4, ***P < 0.001). c) Representative ex vivo images of the tumor (T), spleen (S), and kidney (K). d) Representative images of hematoxylin and eosin staining of tumor sections from the mice with or without antibody treatment. The arrows indicate the immune cells that infiltrated toward the tumor cells.

As shown in Figure 7a,b, in vivo imaging data showed that Q‐annexin V fluorescence was quenched in tumors and surrounding tissues before anti‐PD‐1/4‐1BB antibody treatment on day 10, and this finding was comparable with that of PBS‐injected mice. Notably, fluorescence was selectively turned on in Q‐annexin V‐injected tumor tissues four days after immunotherapy. However, annexin V‐Alexa647 fluorescence was strongly observed in tumors and surrounding tissues, independent of anti‐PD‐1/4‐1BB antibody treatment (**Figure** **7**a). As a result, the tumor‐to‐background ratio significantly increased by 3.8‐fold (from 1.1 to 4.2) in Q‐annexin V‐injected mice following anti‐PD‐1/4‐1BB treatment (Figure 7b). Ex vivo images of the organs also showed that the fluorescence of Q‐annexin V was selectively turned on in tumor tissues only after anti‐PD‐1/4‐1BB treatment, while strong fluorescence of annexin V‐Alexa647 was found in tumor tissues and kidneys before and after anti‐PD‐1/4‐1BB treatment (Figure 7c).

Since abundant lymphocyte infiltration was confirmed in anti‐PD‐1/4‐1BB‐treated mice compared with non‐treated mice by H&E staining of the tumor tissue sections (Figure 7d), our data suggest that Q‐annexin V is a reliable and selective probe for the early detection of apoptosis in tumor tissues, following cancer immunotherapy.

## 3 Discussion

Apoptosis is a dynamic biological process with distinct cell‐to‐cell variations; therefore, live‐cell imaging would be the best technique to study apoptotic events. Most of the current methods are unsuitable for kinetic apoptosis detection due to the washing step.

Here, we report Q‐annexin V as a PET‐based biosensor for monitoring apoptosis in real‐time. Its NIR fluorescence is quenched (OFF) due to the PET interaction between the conjugated dye and amino acid quenchers (tryptophan and tyrosine). ATTO655 fluorescence was efficiently quenched in an aqueous solution when tryptophan or tyrosine were added at various concentrations (Figure 1b), indicating that ATTO655 fluorescence quenching and dequenching are sensitive to the distance between the dye and these amino acids, as reported previously.^[^ ^9^, ^10^, ^11^ ^]^ Indeed, significant quenching was obtained at DL values of < 1, confirming that strong fluorescence quenching of the conjugated dye occurred via PET interactions between the dye and amino acid quenchers, not via FRET between the conjugated dyes.

As mentioned above, PET‐based biosensors are useful for assessing small conformational changes in proteins. If there is a change in the distance between the conjugated dyes and amino acid quenchers due to conformational changes in the protein‐dye conjugate, the quenched fluorescence of the dye could be turned on, thereby enabling small changes in protein conformation to be detected.^[^ ^13^, ^15^ ^]^ Therefore, we treated Q‐annexin V with PPLs to determine whether Q‐annexin V binding to PPL turned on the fluorescence of the conjugated dye due to conformational changes in annexin V upon binding. It is well known that Ca2+‐mediated binding of annexin V to an anionic phospholipid membrane induces conformational changes in annexin V.^[^ ^14^, ^15^, ^16^, ^17^, ^18^ ^]^ As expected, the fluorescence of Q‐annexin V became fourfold brighter when treated with PPLs than with PLLs.

In addition, the Q‐annexin V quenching status was maintained at physiological pH (i.e., pH 7.4) and in the presence of HSA for at least 72 h. This is likely due to the zwitterionic properties of the conjugated ATTO655 dye. Fluorophores with a zwitterionic charge are known to exhibit low levels of serum binding.^[^ ^31^ ^]^ Therefore, Q‐annexin V quenching was maintained in the presence of HSA, as its 3D conformation was not disturbed by nonspecific interactions between the conjugated dye and albumin molecules.

Selective fluorescence turn‐on of Q‐annexin V upon binding with apoptotic cells allowed us to visualize and monitor dynamic and topological apoptosis. In other words, we observed that some cells undergoing early apoptosis survived and we identified which cells underwent apoptosis during asymmetric embryonic stem cell division (Movies S1 and S2, Supporting Information). Using 3D imaging, we observed the topological location of the cells undergoing apoptosis in EB differentiation or when treated with anticancer agents (Movies S3 and S4, Supporting Information). This kind of live cell imaging in 3D cell systems could not be achieved by using the always‐on probe annexin V‐Alexa647 conjugate (Figure 5c). We believe that Q‐annexin V can make many experiments simpler and easier; for example, in the high‐throughput screening of patient‐derived tumor cells for optimal patient‐tailored therapy,^[^ ^32^ ^]^ this probe will eliminate the need to prepare multiple repeated cell plates to determine the time‐course effects of drugs.

As mentioned above, the polarity‐sensitive annexin conjugate developed by Langen's group is very useful for real‐time apoptosis imaging; however, its excitation and emission peaks at 488 and 530 nm limit its applications in vivo. NIR fluorophore conjugates are generally preferred for in vivo applications because of their low autofluorescence, tissue absorbance, and light scattering at NIR wavelengths between 650 and 900 nm.^[^ ^33^, ^34^ ^]^ Indeed, the evaluation of annexin protein‐fluorophore conjugates in the cisplatin‐induced AKI animal model demonstrated that the polarity‐sensitive apoptosis imaging probe pSIVA‐IANBD was not appropriate for in vivo imaging of apoptotic events that occur in deeply seated kidney tissues. In addition, although fluorescence signals from the kidneys could be detected in annexin V‐Alexa647‐injected mice, the data in Figure 6e,f show the limitations of this kind of always‐on‐type imaging probe in the detection of apoptotic events in a cisplatin‐induced AKI model. Since the molecular weight of annexin V is 35 936 Da, it could nonspecifically accumulate in the kidneys during urinary excretion.^[^ ^35^, ^36^ ^]^ Despite acquiring fluorescence images at 3 h post‐injection, the fluorescence signals from the annexin V‐Alex647 that remained in the extracellular space of the kidneys was sufficient to interrupt the fluorescence detection of apoptotic events in the injured kidneys (Figure 6e). In contrast, Q‐annexin V fluorescence was quenched in healthy organs, including the kidneys, and during blood circulation (Figure 6c); therefore, the selective fluorescence turn‐on of this probe in the injured kidneys resulted in a sixfold increase in the fluorescence intensity of the kidney tissues, enabling the real‐time detection of cisplatin‐induced AKI.

The reliability and selectivity of Q‐annexin V for the real‐time monitoring of apoptotic cells in vivo was further confirmed in cancer immunotherapy using ICIs. The data in Figure 7 demonstrate how the selective fluorescence turn‐on of Q‐annexin V upon its binding with apoptotic cells allows us to visualize and monitor the therapeutic efficacy of antibodies. Furthermore, the annexin V‐Alexa647 conjugates were not reliable for selectively detecting apoptosis in tumor tissues following anti‐PD‐1/4‐1BB treatment, due to undesirable high background signals (Figure 7a–c). Because we found strong fluorescent signals in tumor tissues and an abundant tumor infiltration of lymphocytes in anti‐PD‐1/4‐1BB‐treated mice (Figure 7a–d), we conclude that Q‐annexin V is a useful and reliable probe for the early detection of apoptosis in tumor tissues following cancer immunotherapy.

## 4 Conclusion

Herein, we developed Q‐annexin V as the first OFF‐ON‐type NIR fluorescence probe for real‐time apoptosis imaging. The NIR fluorescence of Q‐annexin V is quenched in the extracellular region, but can be selectively turned on upon its binding to apoptotic cells, thereby enabling the real‐time monitoring of dynamic and topological apoptosis in 2D and 3D cell culture systems. Its potential utility for the real‐time monitoring of apoptotic events in vivo was also confirmed in animal models of cisplatin‐induced AKI and cancer immune therapy.

## 5 Experimental Section

##### Materials

Human recombinant annexin V was purchased from BioVision Inc. (CA, USA). The annexin V‐Alexa Fluor 647 conjugate (*λ*_ex_/*λ*_em_: 650/665) was purchased from ThermoFisher Scientific Inc. (Waltham, MA, USA). Dithiothreitol (DTT), sodium dodecyl sulfate (SDS), 2‐mecaptoethanol (ME), HSA, ATTO655‐*N*‐hydroxysuccinimidyl ester (ATTO655‐NHS ester; *λ*_ex_/*λ*_em_: 663/684), L‐tryptophan, cholesterol, doxycycline (Dox; D9891), cisplatin, gelatin, and 2i (CHIR99021; SML1046 and PD0325901; PZ0162) were purchased from Sigma‐Aldrich (St. Louis, MO, USA). N‐acetyl‐L‐tyrosine was purchased from Tokyo Chemical Industry Co., Ltd. (Tokyo, Japan). All lipids were purchased from Avanti Polar Lipids Inc. (Alabama, USA). Amicon Ultra 0.5 mL centrifugal filters (MWCO: 10 kDa) were purchased from EMD Millipore (Billerica, MA, USA). A Sephadex G25 gel filtration column (PD‐mini Trap G25) was obtained from GE Healthcare (Little Chalfont, UK). DMEM (SH30243.01) was purchased from Hyclone (Logan, UT, USA). LIF (ESG1107) was obtained from Merck Millipore (Darmstadt, Germany). Fetal bovine serum (FBS), *β*‐mercaptoethanol, GlutaMAX, Hoechst33342, non‐essential amino acids (NEAA), and penicillin‐streptomycin were obtained from ThermoFisher, propidium iodide (PI) was from ncbit (Seongnam‐si, Korea), and 4’,6‐diamidino‐2‐phenylindole (DAPI) was purchased from Calbiochem (La Jolla, CA, USA). pSIVA‐IANBD (green fluorescence filter set *λ*_ex_/*λ*_em_: 488/530) was purchased from Novus Biologicals (CO, USA). Anti‐mouse PD‐1 mAb (CD279, RMP1‐14 clone) and anti‐mouse 4‐1BB mAb (CD137, 3H3 clone) were purchased from BioXcell (Lebanon, NH, USA), respectively.

##### Fluorescence Quenching of ATTO655 Dye by Tryptophan and Tyrosine

The fluorescence quenching of free ATTO655 dye by amino acids was evaluated by measuring the fluorescence intensity of the dye in the presence of various tryptophan (Trp) and tyrosine (Tyr) concentrations. Water‐soluble N‐acetyl‐L‐tyrosine was used for the quenching test instead of Tyr because of its limited water solubility. The concentration of free ATTO655 dye was fixed at 1 × 10^−6^ m. The fluorescence intensity (*λ*_ex_ 663 nm, *λ*_em_ 684 nm) of sample solutions with various amino acid concentrations was measured using a Safier II multifunctional microplate reader (Tecan, Männedorf, Switzerland).

##### Q‐Annexin V Synthesis and Characterization

Human recombinant annexin V (0.2 mg, 5.6 µmol) and ATTO655‐NHS esters were dissolved in 0.3 mL of 10 × 10^−3^ m PBS (pH 7.4, 137 × 10^−3^ m NaCl) at molar ratios of 1:1, 1:5, and 1:10, respectively, and reacted for 1 h with gentle shaking at 25 °C. The reaction mixtures were passed through a Sephadex G25 gel filtration column to remove unbound fluorophores and byproducts. The obtained Q‐annexin V was concentrated using Amicon Ultra centrifugal filters and stored at 4 °C before use.

The degree of labeling (DL) of the purified Q‐annexin V was determined by measuring the absorbance of the conjugates in PBS buffer with a UV/Vis spectrophotometer (DU750, Beckman Coulter, Brea, CA, USA). The molar extinction coefficient of annexin V (23380 m ^−1^ cm^−1^ at 280 nm) was calculated using ProtParam. The molar extinction coefficient of ATTO655 (1.25 × 10^5^ m ^−1^ cm^−1^ at 663 nm) was obtained from the manufacturer's website.

The inhibition of the fluorescence emission of the dye in the conjugate was then analyzed. Q‐annexin V with different DL values was diluted in PBS buffer (1 × 10^−6^ m dye equivalent), and the fluorescence spectra of the solutions were recorded using a fluorescence microplate reader (*λ*_ex_ 684 nm). The fluorescence intensity of free ATTO655 dye at 1 × 10^−6^ m was used as an unquenched control (zero DL).

The fluorescence changes in Q‐annexin V and annexin V‐Alexa647 following changes in their 3D structure were evaluated by treatment with a denaturation buffer solution. Q‐annexin V and annexin V‐Alexa647 (0.1 × 10^−6^ m dye equivalent) were diluted in PBS (pH 7.4) and the denaturation buffer (1% SDS + 1 × 10^−3^ m 2‐ME), respectively, and their fluorescence spectra were measured using a microplate reader (*λ*_ex_ 600 nm, *λ*_em_ 610–850 nm).

##### Stability of Q‐Annexin V Quenching

Fluorescence changes in Q‐annexin V were examined at physiological pH and in the presence of serum proteins. Q‐annexin V (1 × 10^−6^ m dye equivalent, DL = 1.3) was incubated in either PBS (pH 7.4) or PBS (pH 7.4) containing 10% HSA. The fluorescence intensity (*λ*_ex_ 663 nm, *λ*_em_ 684 nm) of the solution was measured periodically for 72 h.

##### Fluorescence Recovery upon Binding to PS‐Presenting Phospholipid Liposomes

The fluorescence recovery of Q‐annexin V was evaluated by mixing the conjugate with PS‐rich liposomes. PLLs and PPLs were prepared in 10 × 10^−3^ m HEPES buffer (pH 7.4, 2.5 × 10^−3^ m CaCl_2_). To prepare PPLs, 1‐palmitoyl‐2‐oleoyl‐sn‐glycero‐3‐phosphocholine (16:0‐18:1 POPC), 1‐palmitoyl‐2‐oleoyl‐sn‐glycero‐3‐phospho‐L‐serine (sodium salt, POPS), and cholesterol were dissolved in chloroform at a 4:1:1.33 molar ratio in a round‐bottomed flask and dried under argon gas. PLLs were prepared by mixing 1‐palmitoyl‐2‐oleoyl‐sn‐glycero‐3‐phosphocholine (16:0‐18:1 PC) and cholesterol at a 1.5:1 molar ratio, followed by the same method as for the PPLs.^[^ ^21^ ^]^ Dried lipids were placed in a freeze drier overnight to remove the remaining chloroform. HEPES buffer solution (10 × 10^−3^ m, pH 7.4) containing 2.5 × 10^−3^ m CaCl_2_ was added to the flask, vigorously vortexed, and subjected to five freeze‐thaw cycles in liquid nitrogen and warm water baths. Fully hydrated lipid suspensions were extruded through a 1 µm membrane filter using a Mini‐Extruder (Avanti Polar Lipids Inc.; Alabaster, Alabama, USA). The hydrodynamic size and surface charge of the prepared liposomes were measured using a Zetasizer (Malvern Instruments, Malvern, UK).

PPLs and PLLs were mixed with various concentrations of Q‐annexin V and annexin V‐Alexa647. After 15 min of reaction at 25 °C, the fluorescence intensity of the annexin V‐dye conjugates (*λ*_ex_ 620 nm, *λ*_em_ 665 nm) was measured using a fluorescence microplate reader.

##### Cell Culture and Chemicals

ZHBTc4 cells were kindly provided by Hitosi Niwa (RIKEN, Japan) and maintained as described previously,^[^ ^22^ ^]^ with some modifications. Briefly, cells were cultured in 0.1% gelatin‐coated tissue culture dishes in LIF‐2i‐ESC culture medium containing DMEM supplemented with LIF (1000 U mL^−1^ ESGRO), 2i (CHIR99021 3 × 10^−6^ m and PD0325901 1 × 10^−6^ m), FBS (15 v/v %), GlutaMAX (2 × 10^−3^ m), NEAA (1 v/v %), *β*‐mercaptoethanol (55 × 10^−6^ m), penicillin (100 U mL^−1^), and streptomycin (100 µg mL^−1^). To reduce Oct4 expression, doxycycline (Dox, 2 × 10^−6^ m) was added for the indicated times. EBs were formed as previously described.^[^ ^24^ ^]^ H358 human lung cancer cells were purchased from the Korean Cell Line Bank (KCLB, Seoul, Korea) and cultured as tumor spheroids (TS), as described previously.^[^ ^37^ ^]^

##### Flow Cytometry

ZHBTc4 cells were treated with or without Dox (2 × 10^−6^ m) for 3 days and stained with fluorescein isothiocyanate (FITC)‐conjugated annexin V (*λ*_ex_ 488 nm, *λ*_em_ 515–545 nm) and PI (*λ*_ex_ 488 nm, *λ*_em_ 549–601 nm) using the annexin V kit (BD Biosciences; San Jose, CA, USA), as described previously.^[^ ^38^ ^]^ Samples were analyzed at the Flow Cytometry Core Facility (National Cancer Center) using a FACSVerse (BD Biosciences).

##### Confocal Microscopy

Confocal microscopy was performed as described previously.^[^ ^39^ ^]^ Briefly, cells were seeded in a Lab Tek II 8‐chamber (Thermo Scientific) and treated with or without Dox for 3 days. Just before imaging, Q‐annexin V (1 × 10^−6^ m dye equivalent), PI (3 µg mL^−1^), and DAPI (2 µg mL^−1^) were added to the cell culture media. Confocal images (*λ*_ex_ 633 nm, *λ*_em_ 647–754 nm for Q‐annexin V; *λ*_ex_ 488 nm, *λ*_em_ 566–718 nm for PI; *λ*_ex_ 405 nm, *λ*_em_ 415–509 nm for DAPI) were obtained at the Microscopy Core Facility (National Cancer Center) on a LSM510 META (Carl Zeiss, Jena, Germany).

##### Real‐Time Imaging of Apoptotic Cells

ZHBTc4 cells cultured in the Lab Tek II 8‐chamber were treated with or without Dox for 2 days. After adding Q‐annexin V (0.5 × 10^−6^ m dye equivalent) and PI (3 µg mL^−1^), cells were imaged at 30 min intervals for 3 days in real time without washing. Real‐time confocal images (*λ*_ex_ 633 nm, *λ*_em_ 647–754 nm) were obtained at the Imaging Core Facility (National Cancer Center) on an LSM 780 instrument (Carl Zeiss, Jena, Germany).

##### Application to 3D Cell Cultures

EBs derived from ZHBTc4 cells were transferred to Cell Tak (#354 240, Corning; Corning, NY, USA)‐coated Lab Tek II 8‐chambers and Q‐annexin V (0.5 × 10^−6^ m dye equivalent), PI (3 µg mL^−1^), and DAPI (2 µg mL^−1^) were added. H358 tumor spheroids were transferred to Cell Tak‐coated Lab Tek II 8‐chambers, treated with cisplatin (20 × 10^−6^ m) for 24 h, and Q‐annexin V (1 × 10^−6^ m dye equivalent), PI (3 µg mL^−1^), and Hoechst33342 (35 × 10^−6^ m; *λ*_ex_ 405 nm, *λ*_em_ 410–488 nm) were added. In both cases, a few minutes after the probes were added, Z‐stack images were obtained, and 3D images were obtained by confocal microscopy (LSM 510 META; Carl Zeiss, Jena, Germany). Surface rendered 3D images were obtained using the imaris (Bitplane). For comparison, H358 tumor spheroids treated with cisplatin were also stained with annexin V‐Alexa647 (1 × 10^−6^ m dye equivalent; *λ*_ex_ 633 nm, *λ*_em_ 656–755 nm), PI (3 µg mL^−1^), and Hoechst33342 (35 × 10^−6^ m). A few minutes after the probes were added, Z‐stack images were obtained, and 3D images were constituted by confocal microscopy.

##### In Vivo Imaging of Cisplatin‐Induced AKI

All animal studies were reviewed and approved by the Institutional Animal Care and Use Committee of the National Cancer Center Research Institute (NCC‐20‐525), an Association for Assessment and Accreditation of Laboratory Animal Care International accredited facility. Female athymic nude mice (BALB/c‐nu, 5 weeks old) were purchased from OrientBio (Seongnam‐si, South Korea). To generate the cisplatin‐induced AKI model, BALB/c‐nu mice (*n* = 9) received an intravenous injection of cisplatin (5 mg kg^−1^) once a day for three days.^[^ ^28^ ^]^ After 24 h from the third drug injection, annexin protein‐fluorophore conjugates (i.e., pSIVA‐IANBD, Q‐annexin V, and annexin V‐Alexa647) were intravenously injected into the mice (*n* = 3 per group) at a dose of 5 nmol annexin protein equivalent/0.1 mL of PBS. For comparison, a separate set of mice that did not receive cisplatin treatment was administered intravenous annexin V‐fluorophore conjugate injection at the same dose (*n* = 3 per group) and PBS (0.1 mL). Then, fluorescence images were obtained 3 h after the injection of the conjugates using the IVIS system (*λ*_ex_ 460/20 nm and *λ*_em_ 570/40 nm for pSIVA‐IANBD; *λ*_ex_ 640/20 nm and *λ*_em_ 710/40 nm for Q‐annexin V and annexin V‐Alexa647). For ex vivo images, the spleen, kidney, and liver were collected from the mice, and fluorescence images were obtained under the same conditions.

Cisplatin‐induced acute kidney damage was verified by H&E staining of the tissue sections. H&E staining images of the tissue slides were visualized using a Vectra Polaris automated quantitative pathology imaging system (PerkinElmer, MA, USA).

##### In Vivo Apoptosis Imaging in an MC38 Syngeneic Tumor Model

Female C57BL/6 mice (6 weeks old) were purchased from OrientBio (Seoul, Korea). Then, MC38 tumor cells (5 × 10^4^ cells/0.1 mL) were subcutaneously implanted into their right shoulders, and tumor growth was monitored daily. When the tumor size reached ≈100 mm^3^, anti‐PD‐1 plus anti‐4‐1BB (0.1 mg/0.1 mL PBS per injection) antibodies were injected into tumor‐bearing mice on days 10 and 12. On day 14, Q‐annexin V or annexin V‐Alexa647 was intravenously injected into the mice (*n* = 4 per group) at a dose of 5 nmol annexin V equivalent/0.1 mL PBS. Then, in vivo NIR fluorescence images were obtained after 3 h of the injection of the conjugates using an IVIS system (*λ*_ex_ 640/20 nm, *λ*_em_ 710/40 nm). For comparison, a separate set of tumor‐bearing mice received intravenous injections of PBS (0.1 mL; *n* = 4), Q‐annexin V (5 nmol annexin V equivalent/0.1 mL PBS; *n* = 4), and annexin V‐Alexa647 (5 nmol annexin V equivalent/0.1 mL PBS; *n* = 4) on day 10 after tumor implantation, to analyze the fluorescence signals in the tumor tissues in the absence of antibody treatment. Then, NIR fluorescence images were obtained 3 h after the injection of the conjugates. Thereafter, ex vivo NIR fluorescence images of the tumor, spleen, and kidney were obtained.

H&E staining of the tumor sections was performed to verify the antitumor activity of the immunotherapy.

##### Statistical Analysis

Data are presented as the mean ± standard deviation (SD). Student's *t*‐test was performed to determine significant differences between test groups. Differences of *P* < 0.05 were considered significantly different from the control. SigmaPlot (Systat Software, Inc., CA, USA) was used for statistical analysis.

## Conflict of Interest

The authors declare no conflict of interest.

## Supporting information

Supporting Information

Supplemental Movie 1

Supplemental Movie 2

Supplemental Movie 3

Supplemental Movie 4

Supplemental Movie 5

## References

[1] P. Meier , A. Finch , G. Evan , Nature 2000, 407, 796. 11048731 10.1038/35037734

[2] B. A. Carneiro , W. S. El‐Deiry , Nat. Rev. Clin. Oncol. 2020, 17, 395. 32203277 10.1038/s41571-020-0341-y PMC8211386

[3] I. Elia , M. Rossi , S. Stegen , D. Broekaert , G. Doglioni , M. van Gorsel , R. Boon , Escalona‐ , C. Noguero , S. Torrekens , C. Verfaillie , E. Verbeken , G. Carmeliet , S. M. Fendt , Nature 2019, 568, 117. 30814728 10.1038/s41586-019-0977-x PMC6451642

[4] J. Drost , H. Clevers , Nat. Rev. Cancer 2018, 18, 407. 29692415 10.1038/s41568-018-0007-6

[5] D. M. Pardoll , Nat. Rev. Cancer 2012, 12, 252. 22437870 10.1038/nrc3239 PMC4856023

[6] M. S. oldberg , Nat. Rev. Cancer 2019, 19, 587. 31492927 10.1038/s41568-019-0186-9

[7] B. Gabriela , C. Sheridan , S. J. Martin , Methods 2008, 44, 235. 18314054 10.1016/j.ymeth.2007.11.010

[8] Y. E. Kim , J. Chen , J. R. Chan , R. Langen , Nat. Methods 2010, 7, 67. 19966809 10.1038/nmeth.1405 PMC2846705

[9] S. Doose , H. Neuweiler , M. Sauer , Chem. Phys. Chem. 2005, 6, 2277. 16224752 10.1002/cphc.200500191

[10] N. Marme´ , J. P. Knemeyer , M. Sauer , J. Wolfrum , Bioconjugate Chem. 2003, 14, 1133. 10.1021/bc0341324 14624626

[11] S. E. Mansoor , M. A. Dewitt , D. L. Farrens , Biochemistry 2010, 49, 9722. 20886836 10.1021/bi100907m PMC3938424

[12] https://www.atto-tec.com/product_info.php?info=p115_atto-655.html (accessed: January 2020).

[13] H. Kim , H. S. Choi , S. K. Kim , B. I. Lee , Y. Choi , Theranostics 2017, 7, 952. 28382167 10.7150/thno.16647 PMC5381257

[14] J. S.‐D. O. Santosa , M. Vincent , S. Tabariesc , A. Chevalierc , D. Kerboeuf , F. Russo‐Marie , A. Lewit‐Bentley , J. Gallay , FEBS Lett. 2001, 493, 122. 11287008 10.1016/s0014-5793(01)02285-2

[15] L. Silvestro , P. H. Axelsen , Biochemistry 1999, 38, 113. 9890889 10.1021/bi981289o

[16] J. S.‐D. O. Santosa , M. Vincent , M. Takahashi , A. Lewit‐Bentley , J. Gallay , Biochemistry 1999, 38, 5447. 10220332 10.1021/bi982760g

[17] F. Wu , C. R. Flach , B. A. Seaton , T. R. Mealy , R. Mendelsohn , Biochemistry 1999, 38, 792. 9888820 10.1021/bi9819677

[18] A. Follenius‐Wund , E. Piémont , J. M. Freyssinet , D. Gérard , C. Pigault , Biochem. Biophys. Res. Commun. 1997, 234, 111. 9168971 10.1006/bbrc.1997.6596

[19] Y. Zhang , S. Yuan , R. Lu , A. Yu , J. Phys. Chem. B 2013, 117, 7308. 23721323 10.1021/jp404466f

[20] A. Sharma , J. Enderlein , M. Kumbhakar , J. Phys. Chem. Lett. 2017, 8, 5821. 29125301 10.1021/acs.jpclett.7b02430

[21] J. Lu , A. P. Le Brun , S. H. Chow , T. Shiota , B. Wang , T. W. Lin , G. S. Liu , H. H. Shen , Eur. Biophys. J. 2015, 44, 697. 26271933 10.1007/s00249-015-1068-z

[22] H. Kim , H. Jang , T. W. Kim , B. H. Kang , S. E. Lee , Y. K. Jeon , D. H. Chung , J. Choi , J. Shin , E. J. Cho , H. D. Youn , Stem Cells 2015, 33, 2699. 26059508 10.1002/stem.2073

[23] H. Niwa , J. Miyazaki , A. G. Smith , Nat. Genet. 2000, 24, 372. 10742100 10.1038/74199

[24] H. Jang , T. W. Kim , S. Yoon , S. Y. Choi , T. W. Kang , S. Y. Kim , Y. W. Kwon , E. J. Cho , H. D. Youn , Cell Stem Cell 2012, 11, 62. 22608532 10.1016/j.stem.2012.03.001

[25] S. Li , D. Harrison , S. Carbonetto , R. Fassler , N. Smyth , D. Edgar , P. D. Yurchenco , J. Cell Biol. 2002, 157, 1279. 12082085 10.1083/jcb.200203073 PMC2173546

[26] S. Manohar , N. Leung , J. Nephrol. 2018, 31, 15. 28382507 10.1007/s40620-017-0392-z

[27] R. Oun , Y. E. Moussa , N. J. Wheate , Dalton Trans. 2018, 47, 6645. 29632935 10.1039/c8dt00838h

[28] S. J. Holditch , C. N. Brown , A. M. Lombardi , K. N. Nguyen , C. L. Edelstein , Int. J. Mol. Sci. 2019, 20, 3011. 10.3390/ijms20123011 PMC6627318 31226747

[29] P. Avci , A. Gupta , M. Sadasivam , D. Vecchio , Z. Pam , N. Pam , M. R. Hamblin , Semin. Cutaneous Med. Surg. 2013, 32, 41. PMC4126803 24049929

[30] S. Chen , L. F. Lee , T. S. Fisher , B. Jessen , M. Elliott , W. Evering , K. Logronio , G. H. Tu , K. Tsaparikos , X. Li , H. Wang , C. Ying , M. Xiong , T. VanArsdale , J. C. Lin , Cancer Immunol. Res. 2015, 3, 149. 25387892 10.1158/2326-6066.CIR-14-0118

[31] H. S. Choi , S. L. Gibbs , J. H. Lee , S. H. Kim , Y. Ashitate , F. Liu , H. Hyun , G. Park , Y. Xie , S. Bae , M. Henary , J. V. Frangioni , Nat. Biotechnol. 2013, 31, 148. 23292608 10.1038/nbt.2468 PMC3568187

[32] J. K. Lee , Z. Liu , J. K. Sa , S. Shin , J. Wang , M. Bordyuh , H. J. Cho , O. Elliott , T. Chu , S. W. Choi , D. I. S. Rosenbloom , I. H. Lee , Y. J. Shin , H. J. Kang , D. Kim , S. Y. Kim , M. H. Sim , J. Kim , T. Lee , Y. J. Seo , H. Shin , M. Lee , S. H. Kim , Y. J. Kwon , J. W. Oh , M. Song , M. Kim , D. S. Kong , J. W. Choi , H. J. Seol , J. I. Lee , S. T. Kim , J. O. Park , K. M. Kim , S. Y. Song , J. W. Lee , H. C. Kim , J. E. Lee , M. G. Choi , S. W. Seo , Y. M. Shim , J. I. Zo , B. C. Jeong , Y. Yoon , G. H. Ryu , N. K. D. Kim , J. S. Bae , W. Y. Park , J. Lee , R. G. W. Verhaak , A. Iavarone , J. Lee , R. Rabadan , D. H. Nam , Nat. Genet. 2018, 50, 1399. 30262818 10.1038/s41588-018-0209-6 PMC8514738

[33] R. Weissleder , V. Ntziachristos , Nat. Med. 2003, 9, 123. 12514725 10.1038/nm0103-123

[34] E. A. Te Velde , T. Veerman , V. Subramaniam , T. Ruers , Eur. J. Surg. Oncol. 2010, 36, 6. 19926438 10.1016/j.ejso.2009.10.014

[35] J. F. Tait , C. Smith , F. G. Blankenberg , J. Nucl. Med. 2005, 46, 807. 15872355 PMC1201384

[36] H. S. Choi , W. Liu , P. Misra , E. Tanaka , J. P. Zimmer , B. I. Ipe , M. G. Bawendi , J. V. Frangioni , Nat. Biotechnol. 2007, 25, 1165. 17891134 10.1038/nbt1340 PMC2702539

[37] B. Song , D. K. Kim , J. Shin , S. H. Base , H. Y. Kim , B. Won , J. K. Kim , H. D. Youn , S. T. Kim , S. W. Kang , H. Jang , Biochem. Biophys. Res. Commun. 2018, 503, 1980. 30078675 10.1016/j.bbrc.2018.07.145

[38] H. Jang , S. Y. Choi , E. J. Cho , H. D. Youn , Nat. Struct. Mol. Biol. 2009, 16, 910. 19668210 10.1038/nsmb.1657

[39] H. Y. Kim , D. K. Kim , S. H. Bae , H. Gwak , J. H. Jeon , J. K. Kim , B. I. Lee , H. J. You , D. H. Shin , Y. H. Kim , S. Y. Kim , S. S. Han , J. K. Shim , J. H. Lee , S. G. Kang , H. Jang , Exp. Mol. Med. 2018, 50, 137. 10.1038/s12276-018-0166-2 PMC6193020 30333528

