# Supplementary material for: A Quenched Annexin V‐Fluorophore for the Real‐Time Fluorescence Imaging of Apoptotic Processes In Vitro and In Vivo
Source: Adv Sci (Weinh). 2020 Oct 28;7(24):2002988. doi: 10.1002/advs.202002988 (PMC7740095; doi:10.1002/advs.202002988)
Supplement: Supplementary file 1 — Supporting Information [file ADVS-7-2002988-s001.pdf]

© 2020 Wiley-VCH GmbH

ADVANCED SCIENCE
Open Access

Supporting Information

for *Adv. Sci.,* DOI: 10.1002/advs.202002988

A quenched annexin V-fluorophore for the real-time fluorescence imaging of apoptotic processes *in vitro* and *in vivo*

Hyunjin Kim, Hee Yeon Kim, Eun Young Lee, Boem Kyu Choi, Hyonchol Jang, and Yongdoo Choi,**

Supporting Information

A quenched annexin V-fluorophore for the real-time near infrared fluorescence imaging of apoptosis *in vitro* and *in vivo*

Hyunjin Kim, Hee Yeon Kim, Eun Young Lee, Boem Kyu Choi, Hyonchol Jang, and Yongdoo Choi,**

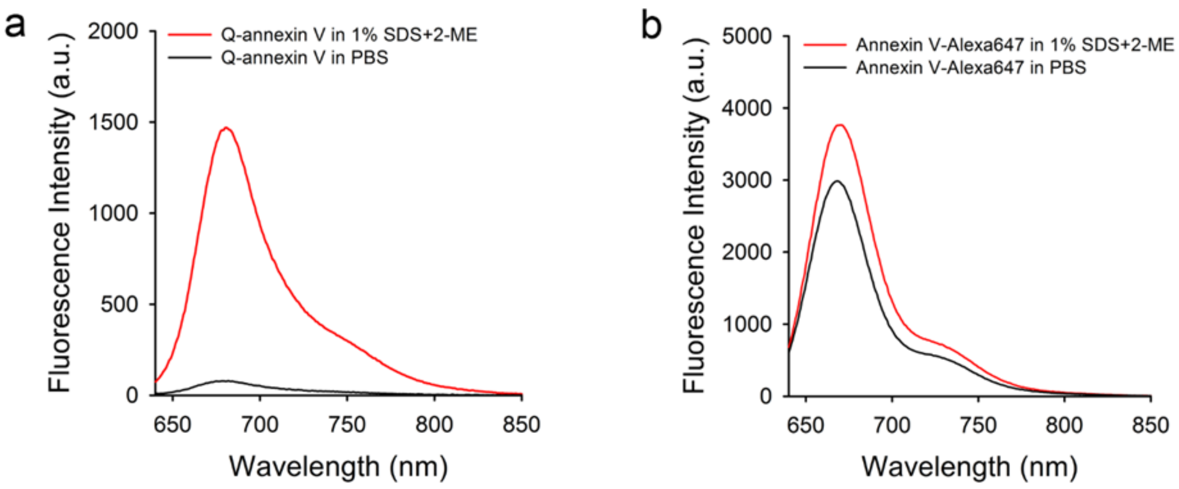

Figure S1. Fluorescence spectra of (a) Q-annexin V and (b) annexin V-Alexa647 conjugates with and without denaturation. Both conjugates were treated with denaturation buffer containing surfactant and reducing agent (1 % SDS + 1 mM 2-ME) to assess fluorescence upon changes in the 3D structure of annexin V. Q-annexin V showed 18.7-fold higher intensity at 684 nm in denaturation buffer than in PBS buffer, whereas a minor increase in fluorescence intensity was observed in annexin V-Alexa647 after denaturation.

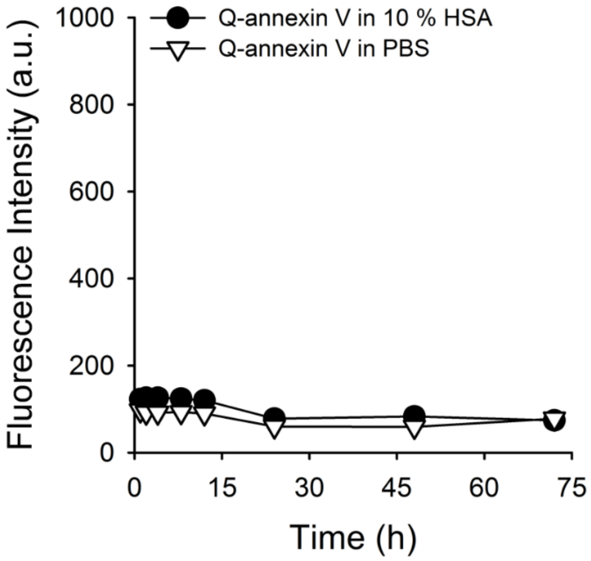

Figure S2. Stability of quenched Q-annexin V at physiological pH and in the presence of human serum albumin (HSA). The fluorescence intensity of Q-annexin V (1 μM dye equivalent) was measured for 72 h.

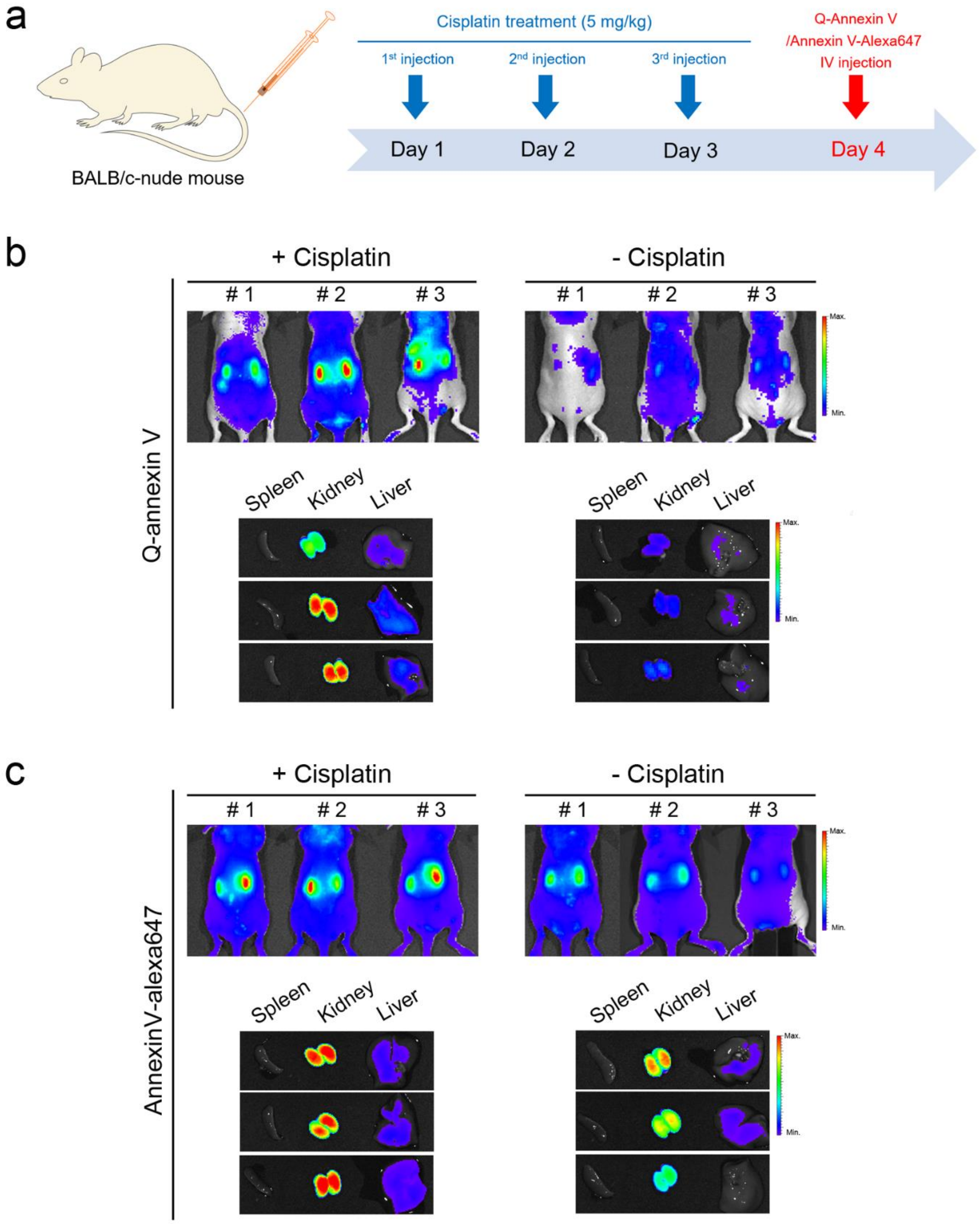

Figure S3. *In vivo* apoptosis imaging in a cisplatin-induced AKI mouse model. (a) A timeline of cisplatin treatment and probe injection. NIR fluorescence images of (b) Q-annexin V- and (c) annexin V-Alexa647-injected mice ($\lambda_{ex.}$ 640/20 nm, $\lambda_{em.}$ 710/40 nm). *Ex vivo* NIR fluorescence images of the spleen, kidneys, and liver are also shown.

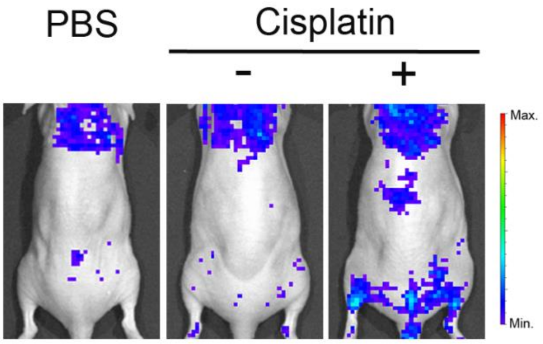

Figure S4. Representative fluorescence images of (a) PBS- and (b) pSIVA-IANBD-treated BALB/c-nu mice.

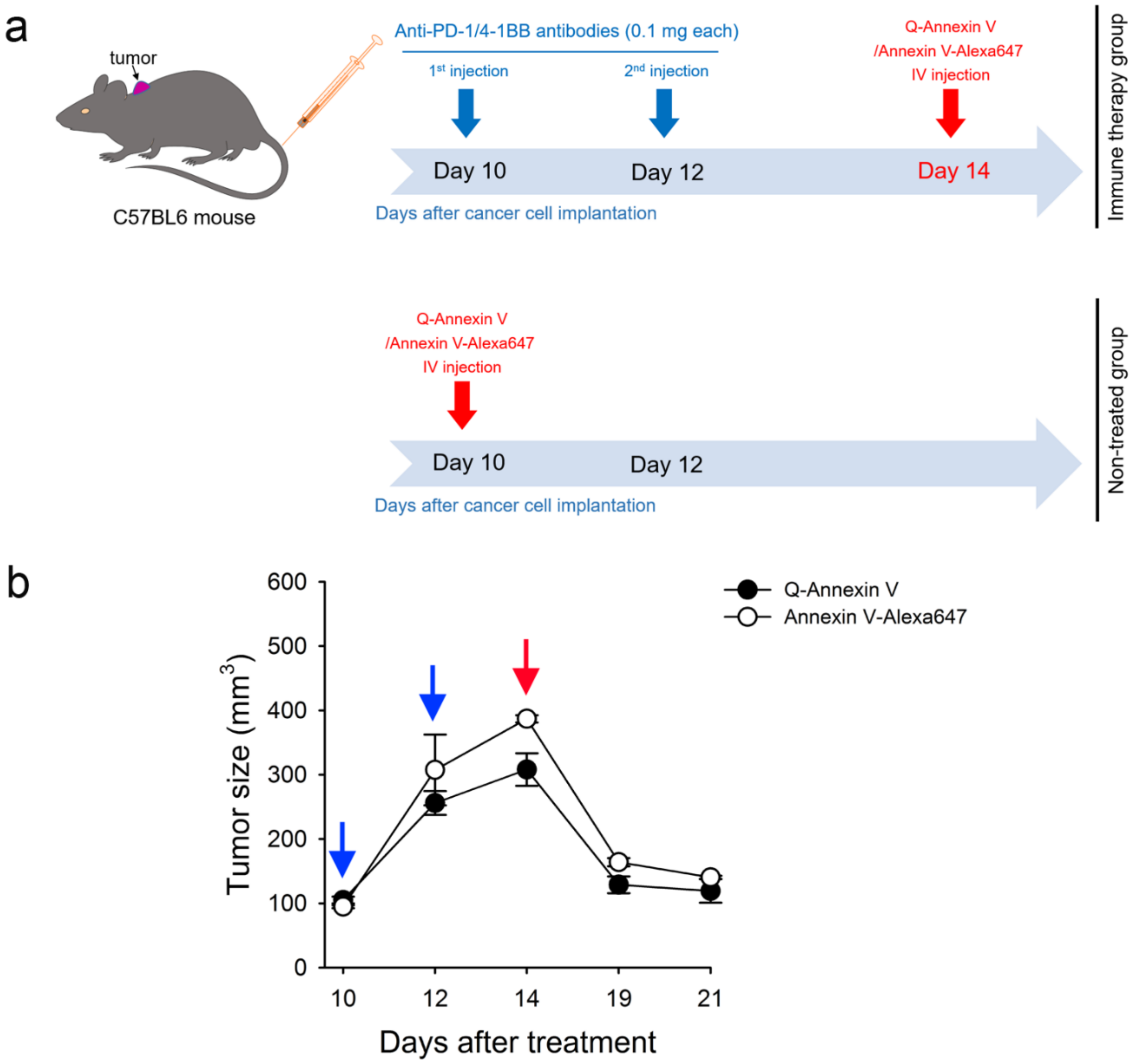

Figure S5. *In vivo* apoptosis imaging in an immune therapy model. (a) A timeline of immune therapy and probe injection. (b) Data regarding tumor growth in the antibody-treated mice. The blue and red arrows indicate the antibody and probe injection time points, respectively.
